# The Consequences of Sexual Harassment in Australian Nightlife Settings

**DOI:** 10.1177/08862605251341281

**Published:** 2025-05-29

**Authors:** Kira Button, Kerri Coomber, Dominique de Andrade, Nicholas Taylor, Eric Koukounas, Zara Quigg, Peter Miller

**Affiliations:** 1 Deakin University, Geelong, VIC, Australia; 2 University of Queensland, Brisbane, Australia; 3 Griffith University, Brisbane, QLD, Australia; 4 Curtin University, Melbourne, VIC, Australia; 5 Liverpool John Moores University, UK

**Keywords:** sexual assault, alcohol and drugs, sexual harassment, adult victims

## Abstract

Sexual harassment and assault are common in Australian nightlife settings; however, the consequences of such harm and factors influencing patrons’ vulnerability to negative outcomes remain under-researched. This study aims to examine (a) the consequences of nightlife-related verbal (e.g., unsolicited sexual comments) and physical (e.g., groping) sexual harassment/assault, as well as pressured sexual contact and (b) the factors associated with these consequences, including demographics, prior experiences of sexual harassment, and frequency of nightlife attendance. Australian adults (*N* = 467; 72% women) who had experienced sexual harassment in nightlife settings in the past 12 months were recruited via social media advertisements and an online panel service (aged 18–64, Mdn = 25, IQR = 22, 29). Participants completed an online survey examining their experiences and consequence of nightlife sexual harassment. Very few participants reported no negative consequences following experiences of verbal harassment (2%), physical harassment/assault (4%), or pressured sexual contact (5%) in a nightlife setting in the prior 12 months. Anxiety (56%–63% range), discomfort (51%–62% range), and anger (44%–52% range) were the most frequently reported consequences. Negative binomial regression analyses found that identifying as a woman or gender-diverse, working as venue staff, and experiencing multiple harassment types was associated with reporting significantly more consequences for verbal sexual harassment. For physical sexual harassment, younger age, infrequent nightlife attendance, and experiencing multiple harassment types were associated with greater consequences, while for pressured sexual contact, only gender was significant, with women reporting more consequences than men. Sexual harassment in nightlife settings is associated with significant adverse emotional and behavioral outcomes, particularly among women, gender-diverse individuals, and those subject to multiple forms of harassment. Preventive interventions must be implemented to reduce nightlife-related sexual harassment and the associated consequences.

Sexual harassment is a global public health concern that places a substantial burden on individuals, communities, and healthcare services (Whetton et al., 2021; World Health Organisation, 2021). While sexual harassment is reported in many public places, research suggests that a disproportionate number of incidents occur in nightlife settings (Mellgren et al., 2018). Nightlife settings are unique as hedonistic behaviors such as excessive alcohol consumption, drug use, and overt sexual behavior are typically normalized (Duff, 2005; Wrightson-Hester et al., 2022). Although nightlife venues primarily attract young adults seeking to have fun, the permissive nature of these environments contributes to them being “hotspots” for sexual harassment (Fileborn, 2012). For instance, patron interviews across several nightlife precincts in Australia found that the prevalence of nightlife sexual harassment ranged from 36% to 56% for women and 14% to 21% for men in the prior 3 months (P. Miller et al., 2016; P. Miller et al., 2019). Despite a substantial proportion of Australian nightlife patrons experiencing sexual harassment, there is a notable gap in our understanding of the consequences of experiencing sexual harassment in these settings (Quigg et al., 2020).

## Defining Sexual Harassment and Assault

Kelly (1987) introduced the concept of the “continuum of sexual violence” to demonstrate that sexual violence extends beyond acts that meet the legal definition of sexual assault. Despite the use of the term “continuum,” this conceptualization does not suggest a hierarchy of sexual behavior but rather highlights how commonplace sexual behavior and conduct (e.g., sexual jokes and comments) can contribute to a broader culture that permits more severe forms of sexual harassment and assault. In nightlife settings, frequently reported incidents of sexual harassment include physical acts such as groping, verbal conduct such as unsolicited sexual comments or jokes, and persistent pressure for sexual contact (Graham et al., 2017; Quigg et al., 2020). It is worth noting that certain physical incidents, such as inappropriate touching of a sexual nature and forced kissing, meet the legal definition for sexual assault in most Australian jurisdictions (Queensland Government, 2018; Victoria Legal Aid, 2023).

## Consequences of Sexual Harassment

Research in university, workplace, and sporting contexts suggests experiencing sexual harassment and assault can result in short and long-term emotional consequences (Bondestam & Lundqvist, 2020; Duque Monsalve et al., 2022; Fasting et al., 2002; Pina & Gannon, 2012; Wolff et al., 2017). A person’s sense of safety and autonomy can be violated through experiences of sexual harassment (Rose, 1986), resulting in varying emotional consequences including anger, discomfort, irritation, sadness, anxiety, self-blame, and feeling helpless or powerless (e.g., Bondestam & Lundqvist, 2020; Pina & Gannon, 2012; Roosmalen & McDaniel, 1999). In more severe cases (e.g., sexual assault) individuals can experience trauma from the incident, and if the trauma is not adequately processed, it may result in lasting consequences (Sigurdardottir & Halldorsdottir, 2021). Experiencing sexual harassment has also been shown to lead to behavioral changes such as increased alcohol use (Kaufman et al., 2019) and avoiding certain places or people (Duque Monsalve et al., 2022; Ferraro, 1996). These behaviors may serve as coping strategies or attempts to prevent revictimization.

A study of primarily female college students found that physical sexual harassment often impaired academic and work performance, while verbal sexual harassment more frequently led to discomfort (Pinchevsky et al., 2020), suggesting that consequences may vary by harassment type. This is supported by earlier work, which also found differences in consequences reported by college women who experienced a range of sexual harassment types including demands for sexual favors in return for a task, unwanted touching, and sexual comments (Roosmalen & McDaniel, 1999). Physical sexual harassment is often perceived as more serious than verbal harassment (Abbey et al., 2004), and may therefore lead to more severe consequences. However, this is disputed by large-scale research on female military personnel, which suggested that there were minimal differences in severity of consequences across harassment types (Langhout et al., 2005). It should be noted that most studies to date have sampled college students (e.g., Kaufman et al., 2019), workers (Pina & Gannon, 2012) and women (e.g., Langhout et al., 2005; Roosmalen & McDaniel, 1999). Consequently, the patterns and types of consequences reported in these studies may not accurately reflect the consequences that arise from sexual harassment in other contexts, or the experiences of male and gender diverse individuals.

## Sexual Harassment in Nightlife Settings

Given the unique social norms and permissive nature of nightlife settings, individual’s experiences of sexual harassment and its impact may be distinct from other contexts. Behaviors that would not be tolerated in other settings (e.g., workplaces), such as excessive alcohol use and overt sexual behavior are typically normalized within nightlife settings (Anitha et al., 2021; Graham et al., 2024; Parks et al., 2021). Many individuals, particularly men, attend nightlife venues with the intention of engaging in sexual behavior or finding a sexual partner (Grazian, 2007; Ronen, 2010). Men commonly use these spaces to initiate sexual encounters with women (Grazian, 2007), reflecting gendered norms that position men as the initiators of sexual contact and women as “sexual gatekeepers,” responsible for accepting or declining advances (Cowley, 2014). Consistent with this, international research suggests that men are the primary perpetrators of nightlife-related sexual harassment (Becker & Tinkler, 2021; Graham et al., 2014).

Although women are expected to manage and reject unwanted advances, research indicates that these refusals are not always respected or accepted. Both patron accounts (Bogren et al., 2024; Fileborn, 2012; Gómez et al., 2022) and observational research (Graham et al., 2010) suggest that some men respond to rejection with continued pressure or escalate to verbal or physical aggression. These norms, behaviors, and attitudes toward sexualized behavior in nightlife settings create ambiguity around consensual and non-consensual behavior. They may lead individuals, particularly women, to minimize harassment, question their own actions, and internalize blame (Anitha et al., 2021; Donde, 2015; Fileborn, 2012). Normative expectations around sexual behavior within nightlife settings can also affect how other patrons and venue staff respond to sexual harassment, often resulting in incidents being minimized or dismissed (Anitha et al., 2021).

## Consequences of Sexual Harassment in Nightlife Settings

Few studies have specifically examined the consequences of sexual harassment within nightlife settings (Quigg et al., 2020). Mellgren et al. (2018) examined female college student’s experiences of sexual harassment in public places and found that 49% reported experiencing sexual harassment in a club, bar, or restaurant. Anger (49%) and worry (21%) were the most common consequences reported, although over 35% of women indicated that they had not been affected (Mellgren et al., 2018). However, as the study did not differentiate consequences across setting (e.g., nightlife, workplace, public transport), the prevalence of nightlife-related sexual harassment consequences remains unclear. Few open-ended responses detailed specific consequences of nightlife sexual harassment, which included participants avoiding venues in which they were harassed and changing their clothing style to be “less revealing” (Mellgren et al., 2018). Similarly, a Canadian study that surveyed (online) female bargoers on the emotional consequences of sexual harassment reported high degrees of negative emotions (e.g., 91% annoyed, 89% uncomfortable) after experiencing unwanted touching (Graham et al., 2017). Stronger negative responses (e.g., anger) occurred more frequently following unwanted sexual touching compared to persistent harassment, although around 50% of participants also reported that they did not take the incident seriously (Graham et al., 2017). It is possible that the sexualized and permissive nature of nightlife venues leads to the minimization of sexual harassment when it does occur (Gunby et al., 2020). Despite these findings, examination of the consequences of sexual harassment within nightlife settings remains limited with regard to the number of studies, type of consequences, and victim characteristics (i.e., only women), highlighting the need for further investigation. In addition, these findings raise questions around the variance in individual responses to sexual harassment; specifically, why some individuals report no or few consequences, while others disclose many negative outcomes, even when experiencing similar forms of harassment.

## Who Is Most at Risk for Experiencing Negative Consequences?

While developing interventions to prevent sexual harassment in nightlife settings is essential (Button et al., 2024), it is also important to consider how to support nightlife patrons who experience sexual harassment. Identifying patrons who are most vulnerable to its negative consequences could inform such support services; however, research in this area is scarce. In workplace settings, frequency of experiencing sexual harassment has been shown to predict negative mood for women, but not men (Nielsen & Einarsen, 2012). Similar patterns have been observed in college settings, where women reported greater impacts on academic performance, physical health, and mental well-being compared to men (Athanasiades et al., 2023), highlighting likely gender differences in the consequences of sexual harassment.

Additionally, adaptive coping strategies (e.g., task-oriented coping) increase from the age of 17 to 25, whereas maladaptive coping strategies (e.g., emotion-oriented coping) are high in early adulthood and decrease over time (Wingo et al., 2015). Consequently, younger adults may be more vulnerable to negative outcomes following sexual harassment due to inadequate coping resources. Similarly, individuals who experience sexual harassment or assault repeatedly or in various forms tend to experience higher rates of negative emotional and behavioral outcomes (Davis et al., 2002; Kaufman et al., 2019; Mellgren et al., 2018), suggesting a cumulative impact of sexual harassment on increasing adverse consequences. Furthermore, it is possible that frequent exposure to sexual harassment may heighten individuals’ sensitivity to its negative effects. Frequent nightlife attendees and venue workers who are likely to regularly witness or experience harassment (Green, 2022; Quigg et al., 2024) could become more susceptible to its adverse consequences over time. Collectively, these findings indicate that negative consequences resulting from sexual harassment may be more frequent or severe in some high-risk groups.

## The Current Study

Approximately one in every two women and one in every five men who participated in patron surveys in Australian nightlife settings report experiencing sexual harassment (P. Miller et al., 2016; P. Miller et al., 2019). Alcohol consumption is deeply embedded in Australian social culture, with young people experiencing both implicit social expectations and explicit peer pressure to consume alcohol, particularly in group settings (Hepworth et al., 2016). Rates of binge drinking and pre-drinking among Australian young adults are also particularly high (Anderson et al., 2020; Degenhardt et al., 2013). Together, these patterns may not only increase the likelihood of sexual harm occurring in Australia but also influence how its consequences are experienced. Despite this, there remains a limited understanding of the impact that nightlife-related sexual harassment has on individuals or the factors that contribute to a person’s vulnerability to negative consequences. This exploratory study aims to address the gaps in existing literature by answering the following research questions:

1. What are the emotional and behavioral consequences of experiencing verbal sexual harassment, physical sexual harassment/assault, and pressured sexual contact within Australian nightlife settings?
2. How do demographic characteristics, prior experiences of sexual harassment, and nightlife attendance relate to the number of consequences reported from nightlife-related sexual harassment?

## Method

### Procedure

Ethics approval was granted by the Deakin University Human Research Ethics Committee. Firstly, participants were recruited using a social media snowballing sampling method. The research team shared a flyer and survey link across Facebook, Twitter, and Instagram, encouraging users to disseminate the survey with their followers. In addition, paid Meta advertisements (AU$500) were utilized to increase reach. Those who clicked the survey link were redirected to Qualtrics, where they were required to read a plain language statement and provide informed consent to participate in the 20-minute survey. Following survey completion, participants could opt-in to enter the draw to win 1 of 10 $50 online gift cards. Social media recruitment took place from January 2023 to September 2023. Due to the small sample size and limited male participants recruited from social media, an additional sample was recruited directly via the Qualtrics panel provider service. The research team requested a sample of 300 males and 150 females. The Qualtrics team managed the recruitment process, data collection, and participant compensation. Panel recruitment was conducted between September and October 2023, with the total cost per participant amounting to $10.86 (for 460 full responses). To be eligible to participate, participants were required to be 18 years of age or older and have visited an Australian nightlife venue (i.e., pub, club or bar) at least once in the previous 12 months.

### Participants

For the wider study, 500 participants were recruited from the social media recruitment methods. The Qualtrics panel provider service supplied an additional 460 complete responses, resulting in a total sample of 960 participants. However, for the current study, only participants who had reported experiencing at least one type of nightlife-related sexual harassment were included (*N* = 467). The sample consisted of 334 females (71.5%), 107 males (22.9%), 18 non-binary individuals (3.9%), 4 people who identified as transgender (0.9%), 2 people who preferred to self-describe (0.4%) and 2 people who preferred not to self-describe (0.4%). Participants’ ages ranged from 18 to 64 (Mdn = 25, IQR = 22, 29).

### Measures

The survey measures utilized in the current study were modified from a tool originally designed to assess risky sexual behavior among female bar drinkers (Parks et al., 2009). The tool was adapted to be inclusive of male and gender-diverse participants and modified to assess additional types of sexual harassment, as well as the consequences of sexual harassment. Although the survey included several sections, the specific items utilized in the current study are detailed below.

### Demographics

Age was included as a continuous variable. Postcode data were utilized to classify participants’ socio-economic status (SES). Postcodes were coded using the 2021 Socio-Economic Index for Areas (SEIFA) Index of Relative Socio-Economic Advantage and Disadvantage (IRSAD; Australian Bureau of Statistics, 2023). Lower SEIFA-IRSAD scores represent higher levels of relative disadvantage. The national SEIFA-IRSAD rankings were divided into tertiles, categorizing participants into low, mid, and high SES. Participants were also asked “What is your gender?” (male, female, transgender, non-binary, prefer not to self-describe, prefer to self-describe). Due to low participant numbers in each of the gender-diverse categories, gender was recoded into (1 = *male*, 2 = *female*, 3 = *gender diverse* [encompassing all other gender categories]). Although the authors recognize the “gender diverse” category is not homogenous, it was included to capture a broader range of gender identities beyond the male and female dichotomy typically examined in nightlife research (Quigg et al., 2020).

### Frequency of Nightlife Attendance

Participants were asked “Over the past 12 months, on average, how often did you visit an Australian nightlife venue (i.e., pub, club or bar)?” Participants chose from eight response options, ranging from “never” to “everyday.” Those who selected “never” were excluded from participating in the survey, and the remaining responses were recoded into four categories: 1 = *a few times per year*, 2 = *once a month*, 3 = *2 to 4 times per month*, 4 = *2+ times per week*.

### Sexual Harassment

Participants were asked about their experiences of three distinct forms of nightlife-related sexual harassment in the past 12 months. Only participants who had experienced at least one of the following forms of sexual harassment were included in the study.

#### Verbal Harassment

Participants were asked “While you were in a nightlife setting, or immediately after leaving a nightlife setting, have you been verbally harassed in the last 12 months?” Affirmative respondents were then asked, “Did you experience the verbal harassment in a sexual (e.g., inappropriate sexual jokes, unsolicited requests for sexual favors) or aggressive manner?” For the current study, only data from participants reporting verbal harassment in a sexual manner were included. A dichotomous verbal harassment variable was created (0 = *did not experience*, 1 = *did experience*).

#### Physical Harassment and Assault

Participants were asked “While you were in a nightlife setting, or immediately after leaving a nightlife setting, have you been physically harassed in the last 12 months?” Affirmative respondents were then asked, “Did you experience the physical harassment in a sexual (e.g., pinching of the bottom, groping, fondling, kissing) or aggressive manner?” For the current study, only data from participants reporting physical harassment in a sexual manner were included. A dichotomous physical harassment and assault variable was created (0 = *did not experience*, 1 = *did experience*).

#### Pressured Sexual Contact

Participants were asked “In the past 12 months, have you experienced someone continually arguing or pressuring you into sexual contact in a nightlife setting or immediately after leaving a nightlife setting when you didn’t want to?” This is distinct from verbal sexual harassment (i.e., which may just be a once off remark), as pressured sexual contact involves persistent, back-and-forth harassment. It is further characterized by a sustained intent to obtain physical sexual contact, an element not always evident in verbal sexual harassment. A dichotomous pressured sexual contact variable was created (0 = *did not experience*, 1 = *did experience*).

### Cumulative Harassment

An ordinal variable was created to account for the number of different types of harassment experienced by participants (1 = *one type*, 2 = *two type*, 3 = *three types*). For example, if a participant had reported experiencing pressured sexual contact, verbal sexual harassment, and physical sexual harassment in the past 12 months, they would be coded as 3.

### Consequences of Sexual Harassment

If participants reported experiencing any type of sexual harassment, they were subsequently asked “Have you experienced or noticed any of the following consequences as a result of being (verbally harassed/physically harassed/pressured into sexual contact) in a sexual manner in the previous 12 months?” Participants were presented with a tick box list of 13 potential emotional (e.g., anger, self-blame) and behavioral (e.g., change in clothing style, lowered alcohol consumption) consequences, along with an option to select “other—please specify” where they could provide a free text response. Participants could select multiple options and also had the option to select “I have not experienced any negative consequences.” Three count variables were created for each participant based on the total number of consequences (0–13) reported for each type of harassment.

### Recruitment Type

As sociodemographic variables can vary across social media and panel samples (Belliveau et al., 2022) a dichotomous variable (0 = *panel*, 1 = *social media*) was created to control for potential differences in the regression analyses.

### Analytical Plan

Frequencies were utilized to determine the percentage of participants who experienced each consequence for each type of harassment. Three negative binomial regressions with log link functions were conducted to determine the relationship between independent variables (e.g., age, gender, SES) and the number of consequences experienced in the past 12 months following each type of sexual harassment. Given the low number of participants, individuals categorized as gender diverse were not included in the physical sexual harassment and pressured sexual contact regression analyses.

## Results

All participants had experienced at least one type of sexual harassment in the previous 12 months, with 334 participants (71.5%) reporting verbal sexual harassment, 243 (41.3%) reporting pressured sexual contact, and 183 (41.3%) reporting physical sexual harassment and assault. Most participants only reported experiencing one type of harassment (55%), followed by two (27.2%) and three (17.8%) types. Table 1 shows the percentage of each consequence experienced by the participants for each type of sexual harassment in the past 12 months. Overall, very few participants reported no negative consequences following an experience of verbal harassment (2%), physical harassment (4%) or pressured sexual contact (5%). Across all types of harassment, anxiety, and discomfort were the most frequently reported consequences, while increasing alcohol consumption and changes in clothing style were the least frequently reported consequences. Some participants described “other” consequences, with responses to verbal sexual harassment including “social seclusion,” “increased-self harm,” “fear of strangers,” and “chopped off my hair—hopefully if I’m less attractive people will leave me the fuck alone.” In response to physical sexual harassment participants reported “confusion,” “avoiding chatting to strangers on nights out,” and “increased severity of mental health issues.” In response to pressured sexual contact, participants expressed a “heightened vigilance,” “unable to relax and have fun,” and “lack of trust toward overly friendly males.”

**Table 1.** Consequences of Sexual Harassment Across Men, Women, and Gender Diverse Participants.

Consequences	Verbal Harassment				Physical Harassment				Pressured Sexual Contact			
	Men (*n* = 53)	Women (*n* = 258)	Gender Diverse (*n* = 23)	Total (*n* = 334)	Men (*n* = 29)	Women (*n* = 140)	Gender Diverse (*n* = 14)	Total (*n* = 183)	Men (*n* = 76)	Women (*n* = 154)	Gender Diverse (*n* = 13)	Total (*n* = 243)
Anger	38%	45%	**52%**	44%	31%	**55%**	**64%**	**52%**	39%	47%	46%	45%
Anxiety	**51%**	**64%**	**78%**	**63%**	**59%**	**61%**	**64%**	**61%**	46%	**62%**	38%	**56%**
Sadness	23%	28%	26%	27%	24%	36%	21%	33%	37%	34%	23%	35%
Irritation	25%	**53%**	**52%**	48%	24%	48%	**64%**	45%	32%	**53%**	**54%**	47%
Helplessness	17%	39%	30%	35%	14%	41%	43%	37%	20%	40%	23%	33%
Discomfort	23%	**67%**	**83%**	**61%**	41%	**65%**	**71%**	**62%**	29%	**60%**	**69%**	**51%**
Self-blame	15%	20%	35%	20%	10%	31%	29%	27%	11%	27%	31%	22%
Decreased self-confidence	15%	26%	39%	25%	24%	25%	29%	25%	11%	24%	23%	20%
Avoiding certain venues/places	17%	**50%**	**57%**	45%	34%	47%	**57%**	46%	20%	39%	31%	33%
Changing body language	17%	31%	**52%**	31%	7%	36%	**50%**	33%	14%	36%	23%	29%
Change in clothing style	13%	17%	22%	17%	14%	24%	29%	22%	3%	21%	23%	16%
Lowered alcohol consumption	11%	27%	17%	24%	21%	32%	7%	28%	12%	30%	8%	23%
Increased alcohol consumption	4%	5%	9%	5%	10%	7%	21%	9%	12%	10%	15%	11%
Other	0%	2%	9%	2%	0%	3%	7%	3%	1%	2%	8%	2%
No consequences	8%	2%	0%	2%	3%	4%	7%	4%	7%	3%	15%	5%

*Note.* As participants could select multiple consequences, percentages add to over 100%. Bolded text indicates a majority.

### Verbal Sexual Harassment

As shown in Table 2, of those who reported verbal sexual harassment in the past 12 months, the number of consequences experienced ranged from 0 to 13 (*M* = 4.8, *SD* = 2.86). The negative binomial regression model for verbal sexual harassment consequences was significant, χ²(12) = 52.83, *p* < .001. Women (incidence rates ratio [IRR] = 1.81) and gender-diverse individuals (IRR = 1.86) reported significantly more consequences than men, and those from high SES areas experienced more consequences than those from low SES areas (IRR = 1.32). Additionally, venue staff (IRR = 1.22) and individuals who experienced all three types of harassment (IRR = 1.29) reported more consequences than those who had not worked in a venue or had only experienced verbal harassment.

**Table 2.** Negative Binomial Regression Models.

Characteristics	Consequences of Verbal Harassment			Consequences of Physical Harassment			Consequences of Pressured Sexual Contact		
	IRR	[95% CI] for IRR	*p*	IRR	[95% CI] for IRR	*p*	IRR	[95% CI] for IRR	*p*
Gender									
Men	Ref			Ref			Ref		
Women	1.81	[1.44, 2.27]	**<.001**	1.27	[0.93, 1.72]	.13	1.50	[1.17, 1.93]	**.002**
Gender diverse	1.86	[1.37, 2.52]	**<.001**	—	—	—	—	—	—
Age	0.99	[0.99, 1.01]	.78	0.98	[0.96, 1.00]	**.02**	0.99	[0.98, 1.01]	.30
SES									
Low SES	Ref			Ref			Ref		
Mid SES	1.21	[0.96, 1.52]	.11	0.97	[0.72, 1.32]	.85	0.92	[0.68, 1.24]	.58
High SES	1.32	[1.04, 1.68]	**.03**	1.11	[0.81, 1.52]	.51	0.98	[0.71, 1.36]	.91
Nightlife attendance frequency									
Infrequently	Ref			Ref			Ref		
Once a month	0.88	[0.73, 1.07]	.15	0.73	[0.55, 0.98]	**.03**	0.96	[0.70, 1.31]	.80
2–4 times per month	0.92	[0.77, 1.10]	.35	0.87	[0.67, 1.12]	.27	1.02	[0.78, 1.34]	.89
2+ times per week	0.82	[0.63, 1.07]	.19	0.73	[0.48, 1.10]	.13	0.7	[0.48, 1.03]	.07
Cumulative harassment									
1	Ref			Ref			Ref		
2	1.06	[0.91, 1.23]	.47	1.26	[0.92, 1.72]	.16	0.86	[0.68, 1.09]	.06
3	1.29	[1.09, 1.51]	**.003**	1.80	[1.32, 2.47]	**<.001**	1.14	[0.91, 1.44]	.21
Nightlife venue worker									
Not a venue worker	Ref			Ref			Ref		
Venue worker	1.22	[1.05, 1.41]	**.009**	1.07	[0.85, 1.34]	.58	0.96	[0.78, 1.19]	.72
Recruitment type									
Panel	Ref			Ref			Ref		
Social media	1.06	[0.91, 1.23]	.43	1.46	[1.15, 1.85]	**.002**	1.29	[1.03, 1.63]	**.03**

*Note. —* indicates no gender-diverse participants in this model. Significant *p*-values are bolded. SES = socio-economic status; CI = confidence interval; IRR = incidence rates ratio.

### Physical Sexual Harassment and Assault

As shown in Table 2, of those who reported physical sexual harassment and assault in the past 12 months, the number of consequences experienced ranged from 0 to 12 (*M* = 5.0, *SD* = 3.21). The regression model for physical sexual harassment consequences was significant, χ^2^(12) = 42.68, *p* < .001. There was a small, significant association between age and consequences, with the expected number of consequences decreasing by 2% for every 1-year increase in age. Further, those who went out once a month experienced fewer consequences than those who only went out a few times per year (IRR = 0.73). Participants who had experienced all three types of sexual harassment reported significantly more consequences than those who had only experienced physical sexual harassment in the past 12 months (IRR = 1.80).

### Pressured Sexual Contact

Of those who reported pressured sexual contact in the past 12 months, the number of consequences experienced ranged from 0 to 13 (*M* = 4.32, *SD* = 3.24). The regression model for consequences experienced following pressured sexual contact was also significant, χ^2^(12) = 45.28, *p* < .001. Recruitment type and gender were the only significant individual predictors in the model, with women reporting significantly more consequences than men (IRR = 1.50).

## Discussion

Many Australians experience sexual harassment within and around nightlife venues, yet little is known about the impact that nightlife-related sexual harassment has on patrons (Quigg et al., 2020). This exploratory study sought to examine the emotional and behavioral consequences of experiencing verbal sexual harassment, physical sexual harassment or assault, and pressured sexual contact within nightlife settings, and how these consequences differ by demographic, prior experiences of sexual harassment and nightlife attendance rate. The study found that almost all individuals who experienced sexual harassment in nightlife settings within the past 12 months reported subsequent negative consequences. Anxiety, discomfort, and anger were the most commonly reported outcomes across verbal sexual harassment, physical sexual harassment, and pressured sexual contact. While the patterns of consequences were somewhat similar across the three types of sexual harassment, differences emerged in the factors that predicted vulnerability to such consequences across harassment types.

The findings from the current study indicate that sexual harassment can have substantial negative emotional ramifications for victims, regardless of the type of harassment experienced. Similar to prior research in workplace and college settings (e.g., Bondestam & Lundqvist, 2020; Pina & Gannon, 2012), anxiety, anger, irritation, and discomfort were commonly reported emotional consequences of nightlife-related sexual harassment. While the prevalence of emotional consequences such as anger and discomfort were slightly lower than those reported in prior nightlife research (Graham et al., 2017), this was likely due to previous research assessing the consequences of the most severe incident that they had ever experienced. Moreover, while 62% of women in college drinking environments attributed “quite a bit” or “total” blame to themselves for being sexually assaulted (Donde, 2015), participants in the current study reported comparatively lower levels of self-blame. This may indicate that self-blame is less prevalent in cases of sexual harassment than assault. Alternatively, the findings may reflect the influence of recent widespread anti-victim blaming campaigns and organizations in Australia (e.g., What Were You Wearing, 2024), leading individuals to attribute responsibility to the perpetrator rather than to themselves. This aligns with Australian police-recorded sexual assault data, which indicates that reporting rates have been steadily increasing, possibly due, at least in part, to growing public awareness and reduced stigma around disclosure (Australian Bureau of Statistics, 2024).

Although behavioral consequences of sexual harassment were reported less frequently than emotional consequences, they remained fairly common among participants. Avoiding certain places or venues was the most frequently reported behavioral consequences following physical and verbal sexual harassment. Similar to statements made by participants in prior research (Mellgren et al., 2018), patrons may feel unsafe in the venue in which the harassment occurred and fear revictimization, which may lead to victims avoiding the venue altogether. This is noteworthy for venue owners and operators, as it is in their best interests to reduce sexual harassment in their venues in order to decrease the likelihood of patrons avoiding their venues. Furthermore, while prior research has suggested that alcohol use may increase following sexual assault (Kaufman et al., 2019), a greater proportion of men and women in the current study reported lowering their alcohol consumption following sexual harassment. As alcohol can inhibit cognitive abilities (Fillmore et al., 2009), lowering alcohol consumption following sexual harassment may be an attempt to increase awareness when in nightlife venues to avoid revictimization. Women reported the highest rates of reduced alcohol consumption, particularly in response to verbal harassment and pressured sexual contact. This may be influenced by the tendency for women to be blamed for their victimization if they were intoxicated (Graham et al., 2024). Double standards around intoxication and blame exist in nightlife settings, whereby alcohol consumption is used to attribute blame to victims while excusing or minimizing the responsibility of perpetrators (Becker & Tinkler, 2015; Graham et al., 2024).

In contrast to prior nightlife research, which identified more severe consequences from physical sexual harassment compared to pressured contact, the current study found comparable consequences across harassment types. As mentioned previously, this discrepancy likely reflects methodological differences, as Graham et al. (2017) assessed the most severe incident whereas the current study examined outcomes of all experiences of sexual harassment in the prior year. Consistent with Langhout et al. (2005), it is likely the pervasiveness of harassment is a stronger predictor of consequences than harassment type. Notably, while overall rates of consequences were comparable across harassment types, gender-specific patterns emerged in the analysis of individual consequences. Consistent with prior research (e.g., Athanasiades et al., 2023), compared to women and gender-diverse individuals, a smaller proportion of men reported experiencing almost every negative outcome. These findings may be partly explained by societal norms around masculinity often discouraging males from expressing their vulnerabilities (Way et al., 2014), potentially making them less likely to report negative consequences. It is also possible that some men experience fewer negative effects due to their lower perceived threat of sexual harassment (Berdahl et al., 1996). One marked gender difference following physical sexual harassment and assault was reported feelings of helplessness, with women and gender-diverse individuals reporting higher rates than men. This could be attributed to the gender power imbalance often prevalent in nightlife settings (Prego-Meleiro et al., 2021). For instance, women and gender minority individuals have reported that rejecting men’s sexual advances can lead to threats or retaliation, including violence (Bogren et al., 2024), which may exacerbate feelings of powerlessness.

Similarly, women and individuals identifying as gender diverse reported higher rates of behavioral consequences (e.g., avoiding venues, changing body language, change in clothing style) following physical and verbal sexual harassment. This may be because women and gender-diverse individuals experience disproportionately higher rates of sexual harassment (P. Miller et al., 2019; Wood et al., 2021), which may lead them to actively adopt protective strategies to minimize further harassment. These behavioral changes may also reflect broader gendered expectations around responsibility for preventing sexual harassment, with the responsibility for rejecting advances typically placed on women (Cowley, 2014; Graham et al., 2024). These patterns of behavioral adaptation are well-documented, with decades of research highlighting how women modify their clothing, appearance, and movement through public spaces in response to the threat of sexual harassment (e.g., Hsu, 2010; Thompson, 1994; Valentine, 1989). For instance, Valentine’s (1989) seminal work highlighted how women’s use of urban space is influenced by concerns about safety, particularly at night.

Notably, very few participants reported experiencing no consequences following sexual harassment. This contrasts with earlier research, which found that 35% of female students who experienced public sexual harassment reported no impact (Mellgren et al., 2018), and approximately half of female bar-goers did not perceive the incident of unwanted touching as serious (Graham et al., 2017). This discrepancy may reflect wider shifts in societal perceptions of sexual harassment over the past decade, as the previous data was collected in 2012 (Graham et al., 2017) and 2013 (Mellgren et al., 2018). Recent social justice movements such as the #MeToo movement resulted in an increased awareness of sexual harassment internationally, reducing both tolerance and stigma around sexual harassment (Gallagher et al., 2019). Consequently, individuals may now be more likely to recognize sexual harassment and articulate the associated negative outcomes.

The current study identified differences in the factors that predicted vulnerability to consequences across harassment types. Gender was a significant predictor for physical and verbal sexual harassment, but not for pressured sexual contact. This may be due to men often perceiving behaviors as sexual harassment only when physical force is used (Osman, 2007), while women are more likely to classify ambiguous behaviors (e.g., invasive comments) as sexual harassment (Prego-Meleiro et al., 2021). Women are also significantly more likely to be worried about victimization in nightlife spaces compared to men (Prego-Meleiro et al., 2021). This pre-existing heightened perception of risk and broader classifications of sexual harassment may result in women experiencing greater consequences. In contrast, pressured sexual contact, which may involve more subtle behaviors such as persistent flirting is often considered to be somewhat flattering by patrons (Graham et al., 2017), potentially resulting in comparable levels of emotional and behavioral consequences across genders. The findings also indicated that younger people were slightly more likely to experience greater consequences of physical sexual harassment, although this association was not found for the other forms of harassment.

For verbal sexual harassment, venue workers experienced more consequences than patrons, although this association was not significant across the other two forms of harassment. Women venue workers have indicated that lewd sexual comments are made toward them on a weekly basis (Green, 2022) and as such may be downplayed or dismissed as a typical part of the job. As a result, for venue workers, the consequences of verbal harassment may go unaddressed and accumulate over time, while physical and persistent incidents might prompt more immediate reactions (e.g., feeling angry or report to the manager), similar to that of patrons. Comparable to prior research (e.g., Mellgren et al., 2018), the current findings also indicated experiencing all three types of sexual harassment resulted in greater consequences following physical and verbal harassment. Experiencing multiple forms of sexual harassment may compound the emotional toll on individuals and also make them more likely to make behavioral changes (e.g., avoiding venues) to prevent future victimization.

### Implications

The findings of the current study highlight the serious nature of sexual harassment within Australian nightlife venues. The high prevalence of negative outcomes among participants underscores the urgent need for implementing evidence-based interventions and policies targeted at preventing sexual harassment within nightlife settings. A scoping review of nightlife sexual harm interventions highlighted that few existing interventions have been evaluated, and even fewer have been shown to be effective in reducing sexual harm in nightlife settings (Button et al., 2024). Further research is needed to inform the development of robust, evidence-based interventions that can reduce the likelihood of sexual harassment occurring, and in turn, lower the risk of associated behavioral and psychological consequences.

The findings of the current study also emphasize the importance of supporting individuals and groups who are most vulnerable to consequences, such as female and gender diverse individuals and those who experience multiple forms of sexual harassment. Reports of sexual harassment are often dismissed by venue staff and security (Anitha et al., 2021) and currently, there are no legal requirements in Australia for venue staff or managers to be trained in recognizing and responding to sexual harassment. Implementing mandatory training could help ensure that reports of sexual from vulnerable groups are taken seriously (Quigg et al., 2021, 2023). It would also ensure patrons are appropriately connected to support services to address the harm caused by such experiences.

### Limitations

The current study has a number of limitations that should be considered. As the research relied on a self-selected sample, there is potential for self-selection bias, which may have influenced the representativeness of the findings. The results may also have been influenced by recall bias, as participants were asked about their experiences of sexual harassment in the previous 12 months. Furthermore, due to low participant numbers, several gender diverse categories (e.g., non-binary, transgender) were grouped together. Given the high prevalence of consequences among this group, future research should explore differences in experiences and outcomes across these individual gender identities. Additionally, this study may have unintentionally excluded more severe forms of sexual harm, such as rape and date rape, due to the way sexual harassment was categorized and the absence of these examples under “physical sexual harassment and assault.” Future research should examine the consequences of severe forms of nightlife sexual harm, given their potential for serious and lasting harm (Ogunwale & Oshiname, 2017). The current study also did not examine the severity of consequences experienced (only number) or distinguish between immediate and long-term consequences of sexual harassment. These facets are important to consider in future research as it could inform the types of support services offered to patrons on the night (e.g., crisis support), as well as longer term interventions (e.g., mental health support services).

## Conclusion

The findings of the current study add to the limited body of evidence on the consequences of nightlife sexual harassment. Experiencing sexual harassment in nightlife settings, irrespective of type, can lead to a broad range of negative outcomes for individuals, including emotional distress and behavior changes. Women and gender-diverse individuals in particular, frequently change their behavior following sexual harassment, possibly in order to prevent future victimization. Given the lack of effective interventions (Button et al., 2024) and the substantial negative consequences identified particularly for high-risk groups, there is an urgent need to address sexual harassment in nightlife venues in Australia. This process should involve implementing strategies to prevent sexual harassment as well as ensuring victims are provided with adequate support if they do experience harm within a venue. Such actions are important in promoting safer nightlife settings and mitigating the adverse impacts for those who experience sexual harassment.
